# Time-Resolved Ion Mobility Spectrometry with a Stop Flow Confined Volume Reaction Region

**DOI:** 10.1021/acs.analchem.4c00434

**Published:** 2024-06-10

**Authors:** Osmo Anttalainen, Markus Karjalainen, Elie Lattouf, Oliver Hecht, Paula Vanninen, Hanna Hakulinen, Tapio Kotiaho, Charles Thomas, Gary Eiceman

**Affiliations:** † VERIFIN, Finnish Institute for Verification of the Chemical Weapons Convention, Department of Chemistry, University of Helsinki, Helsinki FI-00014, Finland; ‡ Airsense Analytics GmbH, Hagenower Straße 73, Schwerin 19061, Germany; § Drug Research Program and Division of Pharmaceutical Chemistry and Technology, Faculty of Pharmacy, University of Helsinki, P.O. Box 56, Helsinki FI-00014, Finland; ∥ Department of Chemistry, Loughborough University, Leicestershire LE11 3TU, U.K.; ⊥ Department of Chemistry, Faculty of Science, University of Helsinki, P.O.Box 55, Helsinki FIN-00014, Finland; # New Mexico State University, 1175 N Horseshoe Dr., Las Cruces, New Mexico 88003, United States

## Abstract

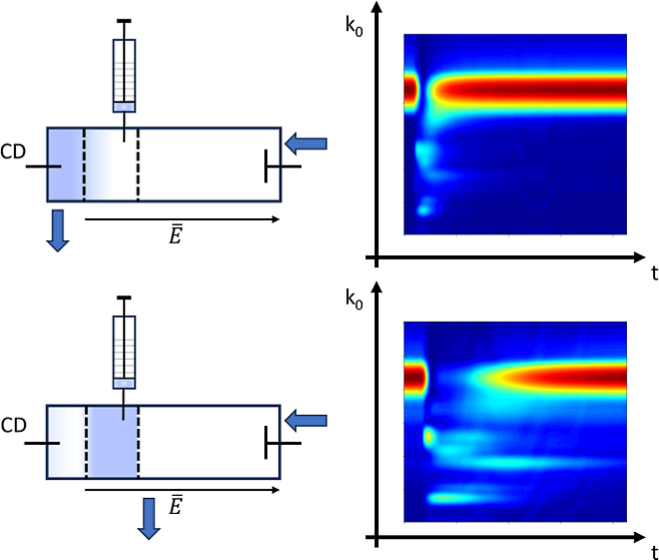

An ion source concept is described where the sample flow is stopped in a confined volume of an ion mobility spectrometer creating time-dependent patterns of ion patterns of signal intensities for ions from mixtures of volatile organic compounds and improved signal-to-noise rate compared to conventional unidirectional drift gas flow. Hydrated protons from a corona discharge were introduced continuously into the confined volume with the sample in air at ambient pressure, and product ions were extracted continuously using an electric field for subsequent mobility analysis. Ion signal intensities for protonated monomers and proton bound dimers were measured and computationally extracted using mobilities from mobility spectra and exhibited distinct times of appearance over 30 s or more after sample injection. Models, and experimental findings with a ternary mixture, suggest that the separation of vapors as ions over time was consistent with differences in the reaction rate for reactions between primary ions from hydrated protons and constituents and from cross-reactions that follow the initial step of ionization. The findings suggest that the concept of stopped flow, introduced here for the first time, may provide a method for the temporal separation of atmospheric pressure ions. This separation relies on ion kinetics and does not require chromatographic technology.

## Introduction

Ion mobility spectrometers have been widely accepted in applications where the chemistry of ion formation is favorable for targeted analytes, including the detection of organophosphorous compounds^1,2^ (OPCs) as chemical warfare agents^3−7^ in military applications, and the determination of explosives^8−11^ during screening of hand-held luggage in airports. In these instances, the high selectivity of ion mobility spectrometry (IMS) can be attributed to the selectivity of ionization of the sample using atmospheric pressure chemical ionization (APCI) with hydrated protons in positive polarity or oxygen anions or chloride ions in negative polarity. Examples include the high enthalpies of association of protons with strong O=P dipoles of organophosphorous compounds^12^ and O_2_^•–^ or Cl^–^ with aromatic nitrate esters.^13^

In some instances, reagents or dopants^14−17^ can be added to the ion source or reaction region to introduce additional selectivity with so-called alternate reactant ions.^18^ In these applications, ion mobility spectrometers can be classified as selective detectors, usually with ppm or ppb detection limits^7^ (or ng cm^–3^ to pg cm^–3^ amounts^19^). When mixtures containing a broad range of ionization properties are introduced directly into APCI-based sources in IMS, findings may not quantitatively show the sample composition. This also may occur when matrix constituents suppress the ionization of the target analyte, creating a false negative response, for targeted analyses^20^ or incomplete inventories with untargeted analyses. Historically, analysis of mixtures by IMS with general response to volatile organic compounds (VOCs) have included preseparation using gas chromatography with examples in air quality monitoring,^21^ analysis of breath metabolites,^22^ chemical characterization of bacteria,^23^ and food quality.^24^ A principal advantage of chromatographic preseparation is the simplification of ionization chemistry since analytes are introduced as individual substances, ideally into the ion source of the drift tube. Alternatives include sample pretreatment with solid phase microextraction,^25,26^ temperature-programmed thermal desorption,^27,28^ or pyrolysis^10^ methods. Disadvantages of such prefractionations include delays in measurements due to chromatographic retention or additional efforts for sample preparation. Regardless of methodology or technology embodiments,^29−32^ the drift tube structures, and management of sample ionization have been largely unchanged since the early 1980s.^33^ A noteworthy exception is the use of reduced pressures to limit competitive charge exchange.^22,32,34^

Residence times for sample neutrals, once introduced into drift gas flows of 100 to 300 mL min^–1^, commonly range from 1 to 8 s as sample is carried through the reaction region volumes of 10 to 40 mL. Such times are relatively long compared to 1 to 100 ms for APCI where reaction times are governed by vapor concentration, reactant ion density, and rate coefficients.^35,36^ Recently, in silico models^37^ showed that product ions were formed with characteristic dependencies for individual VOCs and a binary mixture of VOCs. Computational models were confirmed in laboratory measurements, and such dynamics have been observed in IMS, if not systematically described. The models suggested that primary ionization of constituents in a mixture could be accompanied by subsequent cross-reactions when residence times of sample could be extended. The cross-reactions can stabilize to the most probable ionization product, governed by the highest proton affinity (PA) in the mixture compounds. This process, in principle, may be utilized to separate ions in ionization phase, thus avoiding the need for chromatographic preseparation. Consequently, instrumentation could be simplified, and the detection limits could be improved for compounds otherwise masked with higher concentrations in mixtures.

The objective of this work was to explore time-resolved formation of ions, called gas ion distillation (GID), in an IMS drift tube with a modified unidirectional flow (UDF) design, where user-control vapor residence in a reaction region was achieved with a stop flow confined volume (SFCV) control. In this design, the sample was introduced into a confined volume, and drift gas was extracted before it entered this GID volume or reaction region. Dynamics of ion formation were studied with a ternary mixture and compared to models using a multiphysic finite element method environment (COMSOL multiphysics^38^). The responses for mixtures of VOCs containing up to five constituents were recorded semiquantitatively to document time-resolved ion formation with IMS methods.

## Experimental Section

### Instrumentation

**Figure 1:**
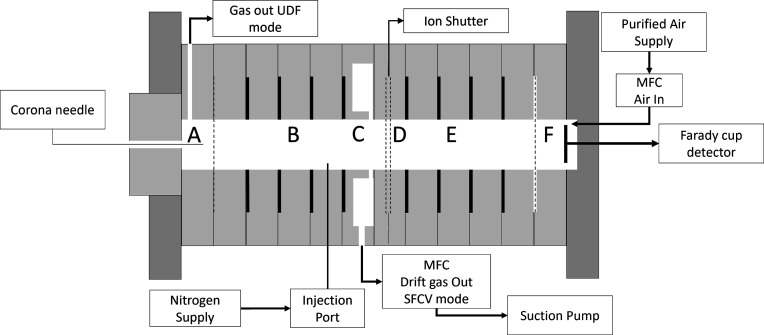
Schematic of ion mobility spectrometer with gas extraction between drift region and enlarged design for ion source. (A) corona ionization and drift gas exit in the UDF-mode, (B) reaction region and sample injection, (C) mass flow controllers (MFC) balanced drift gas exit in SFCV-mode, (D) ion shutter, (E) drift region, and (F) Faraday cup detector and MFC balanced drift gas input. (---) Lines in figure present grids.

An ion mobility spectrometer, based on earlier design,^39^ was prepared using in-house prepared stainless steel drift rings (20 mm inner diameter and 3 mm thick) set in 10 mm thick PTFE rings (Figures 1 and S1).^39^ The ion source was a corona discharge with a point-to-plane design using a needle and mesh as shown in Figure 1. A 13 mm wide teflon ring (C) where drift gas flow could be extracted radially (360°) using a diaphragm pump (SP 625 series, Schwarzer Precision GmbH, Essen, Germany) connected to 1 L flow dampening volume between the ion shutter (D) made using a pair of interdigitated grids as described previously.^39^ Dimensions were 44 mm, reaction region (B) 52 mm, drift region (E), and 3 mm aperture grid to detector (F). A voltage divider was used to set the potential on drift rings as given, with electric fields, in Table S1. Additional details on the complete instrument with a 3D-cut off view of the drift tube are shown in Figure S1.

High voltage for the corona discharge (7 kV) and drift tube (4 kV) was provided by EMCO DC–DC HW (XP Power Ltd., Singapore) converters. The sample was introduced in a small flow (5 mL min^–1^) through a 60 mm length of fused silica capillary tubing connected to a heated injection port (120 °C) from a gas chromatograph (model 5890 Hewlett-Packard, Wilmington, USA). The injection port was held against the drift tube body (98 °C) and the capillary column was protected against cool spots with a glass wool thermal insulator heat band. The capillary entered the reaction region (Section B) via the nearest insulation ring to Section C. Nominal septum purge and vent flows were 4 and 60 mL min^–1^, respectively. Drift gas was house air after treatment in a catalytic air purifier, and tower of 5 Å molecular sieves. Two MFC (Red-y smart meter GSM, Vögtlin Instruments GmbH, Switzerland) were set to matched flow rates (150 mL min^–1^), and they were used to introduce drift gas at the detector and to extract drift gas at point C (Figure 1), under user control.

The drift tube was used in two flow arrangements. When operated as a conventional drift tube, no drift gas flow was extracted at point C, and sample was swept through the ion source with a unidirectional drift gas flow (UDF mode) without any intentional retention of sample. When operated with a stopped flow confined volume (SFCV mode), the sample was extracted at point C under suction with flows that matched the in-flow of drift gas on demand at point C (Figure 1).

### Reagents and Chemicals

Chemicals were chosen to reflect a variety of proton affinities. Chemicals were 6-methyl-5-hepten-2-one (sulcatone) (purity ≥97% PA 875 kJ mol^–1^^40^) and 2-butanone (purity 99%, PA 827 kJ mol^–1^^40^), isopropyl alcohol (purity ≥99.5% PA 793 kJ mol^–1^^40^), and 1-heptanol (purity 98% PA 799 kJ mol^–1^^40^), from Sigma-Aldrich Chemie GmbH (Schnelldorf, Germany), and pentyl acetate (GPR grade PA 840 kJ mol^–1^^41^) from VWR Chemical BDH (Leuven, Belgium). 6-Methyl-5-hepten-2-one was identified to contain octane-2,7-dione (PA 892 kJ mol^–1^^42^) with 2% by mass.^37^ Because of this, the sulcatone -2-butanone mixture may be referred to as a ternary mixture.

## Methods

### Sample Preparation

#### Samples with a Single Constituent

A 1 μL volume of neat liquid sulcatone was injected into a 1 L round-bottom flask kept at 86 °C to ensure evaporation of the constituents using heating tape and insulation. The flask was capped using an adapter with septum. The sample to the vapor in the flask for 10 min, 250 μL was withdrawn using a gastight syringe (250 μL, 5182-9608, Agilent, Australia), and discharged completely. This was repeated, and on a third draw of sample, the volume was reduced to 25 μL. Nitrogen gas, from a continuously purged 1 L flask was pulled into the syringe to 250 μL creating a 1:10 dilution; the volume was reduced again to 25 μL and this process was repeated four times, with a last discharge to 50 μL. These steps produced a 1:10,000 dilution in syringe. A sample of 2-butanone was prepared using an identical procedure. The estimated concentrations in the syringe were 20 and 30 ppbv for sulcatone and 2-butanone, respectively.

#### Samples with Ternary Mixtures

Binary mixtures were prepared following the same approach as described above; 2.5:2.5, 1:1, and 1:7 μL injections of neat liquid sulcatone and 2-butanone into 1 L a heated flask and diluted in the gastight syringe as describe above. The resulting estimated (sulcatone/2-butanone) concentrations after syringe dilution were 50:80, 20:30, and 20:230 ppbv.

#### Multicomponent Mixture

A multicomponent mixture was prepared by mixing 59 μL of sulcatone, 59 μL of pentyl acetate, 57 μL of 1-heptanol, 36 μL of 2-butanone, and 31 μL of isopropyl alcohol in a vial, and injecting 5 μL of this mixture into 1 L a heated flask leading to equal concentration of 25 ppbv of each component after four times syringe dilution. Headspace sample was measured for each component to obtain peak positions for the product ions.

Assuming >10% error estimation for each step of sample preparation, the error based on the parametric sensitivity for sample concentrations is ±22%, and therefore maximum two number accuracy is used to express concentrations.

#### Sample Measurements

A gastight syringe was used to inject 25 or 50 μL sample into the sample region (Figure 1B). Each injection was repeated at least seven times. IMS was operated in two gating modes: for single component and concentration variation experiments, the shutter speed was 500 μs providing high signal-to-noise ratio but low resolving power, and in other experiments, the shutter speed was 250 μs resulting in increased resolving power at the cost of low signal-to-noise ratio. Sample peak intensity variation between maximum and minimum in repetitions was less than 1 V (24% from maximum peak intensity) for samples measured with the 500 μs gate pulse and less than 0.02 V (7.7% from maximum peak intensity) for samples measured with the 250 μs gate pulse. Between the samples, the system was flushed in the UDF-mode until the response had cleared and signal had returned to the baseline, which was ensured by visual inspection of signal level with the data collection software. IMS spectra were recorded with in-house built LabVIEW software (National Instruments, Austin, Texas, Usa) application and amplifiers. In the SFCV mode, drift gas flow into the drift region and gas flow extracted from the GID region were balanced, and like in the UDF mode, IMS was operated at ambient pressure. The sampling rate for ion mobility spectra was 5 μs and a new IMS spectrum was obtained roughly every 100 ms (∼10 Hz). Each such spectrum recorded by the data acquisition system was an average of two consequential spectra. The recorded spectra included time labels for both elapsed time and spectra acquisition points, and these time labels were used in data processing. Recorded data sets were stored in unique files, and laboratory records were kept for traceability.

#### Data Processing

The plots were generated with R-Studio (2023.03.01, Build 446, Posit Software PBC). For plots and data processing, detector response was filtered with 2nd order Butterworth low-pass filter with cutoff frequency of 20 kHz for the data measured with 500 μs gate pulse, and with cutoff frequency of 5 kHz when data were measured with 250 μs gate pulse. The peak positions, intensities, and response times were obtained from spectra with manual assisted in-house-built macros in R-Studio. The inaccuracy of this method in elapsed time is ±0.5 s.

Reduced mobilities were obtained from peak positions using reduced mobility of sulcatone , determined from data provided by Lattouf et al.,^37^ as a reference for other reduced mobilities. The estimated uncertainty of determination of reduced mobilities is . Reduced mobilities for 2-butanone and pentyl acetate were associated only with experimental data, but reduced mobilities for isopropyl alcohol and 1-heptanol are comparable with the data^43,44^ created elsewhere. The zero time in line graphs has been adjusted manually to visualize the moment of sample injection.

#### Computational Modeling

General multiphysic models, including environmental and geometric parameters, were used to simulate the reactions in relevant geometry. The time-resolved ion formation, called here as GID, was studied with two models of the reaction region. First, the hypothetical reaction region arrangement was evaluated in cylinder symmetric geometry (see the Supporting Information, Testing the Separation Hypothesis with Simulation) when the ratio of rate coefficients between two compounds were varied. Second, 2D model was used to study the reactions of ternary mixture in the confined volume reaction region (Supporting Information. Computational Model of Confined Sample). The models included convection and migration of ions by flow and electric field and ion-neutral reactions. To estimate an effect of sample introduction into the concentration distribution in the reaction region, the sample introduction with GC-injector using a capillary column connected to the reaction region was also modeled.

The reaction parameters were selected based on the values given in ref (37) and adjusted for simulation. Rate coefficients for cross-reactions (details in Table S4) were arbitrary selected for the simulation scenario. The ion intensities after the reaction region are averaged over the boundary cross-section (see the geometry in Figure S11). The model includes cross-reactions (Table S4) but not monomer–dimer reaction. The model is also simplified compared to reality since initial vapor concentrations were uniformly distributed in the reaction volume for the SFCV mode and times for mass transport from sample injection are not included. Thus, the model represents the problem under idealized condition. Furthermore, to limit the computational time, the reactant ions were introduced in excess amount to scale and speed up the reactions to <1% of the real experiment time (>100 ms).

## Results and Discussion

### Response to Individual Substances

Response with the GID enhanced ion mobility spectrometer, operated in the UDF mode, for individual substances (M) is shown in Figure S3 for 2-butanone and Figure S4 for sulcatone and arose from reactions in the ion source between reactant ions  and a substance (M). In the absence of the sample, spectra exhibited wide peak of the hydrated protons spread around reduced mobility coefficients between  and . When a substance was introduced to the inlet using a gastight syringe, response occurred <1 s with the formation of protonated monomer , and in most instances, proton bound dimers M_2_H^+^ at increased vapor concentrations through reactions shown in eqs 1 and 2
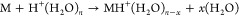
1
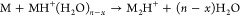
2

**Figure 2:**
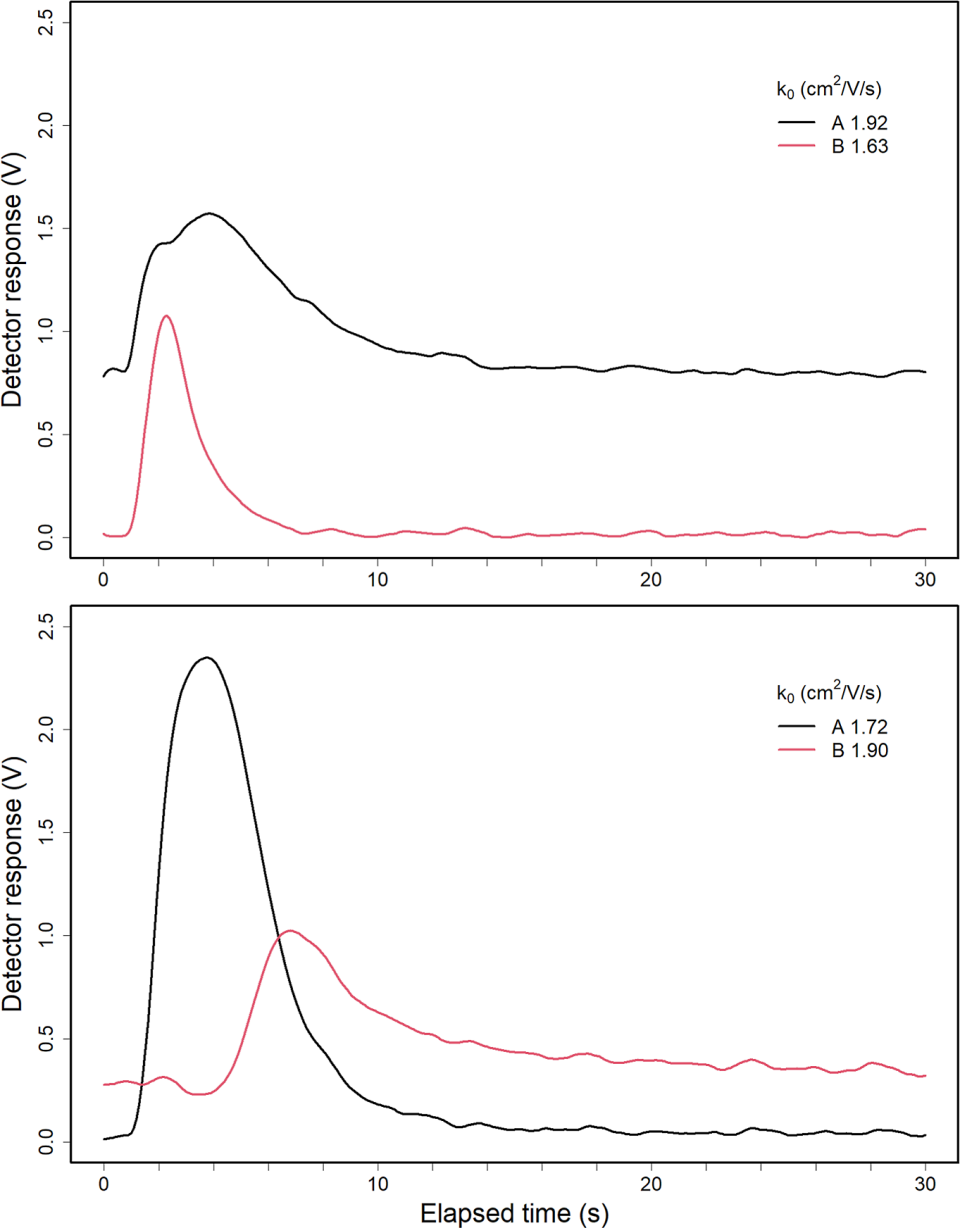
Top frame: 2-butanone sample ion peak intensities over time, when 50 μL at 30 ppbv was injected into the reaction region in the UDF mode. Signals are (A black—) 2-butanone monomer and (B red—) 2-butanone dimer. Bottom frame: the sulcatone with octane-2,7-dione sample ion peaks over time when 20 ppbv of sulcatone sample was injected into the reaction region in the UDF mode. Signals are (A black—) sulcatone monomer and (B red—) octane-2,7-dione monomer. The offsets for 2-butanone monomer and octane-2,7-dione in the figures are caused by overlapping the reactant ion peak (see Figures S3 and S4).

The appearance of ion peaks for these substances is shown in Figures S3 and S4 1 s intervals from 0 to 8 s after an injection. Spectra are consistent with prior IMS measurements,^45^ where the reactant ion is conserved as product ions (i.e., protonated monomers and proton bound dimers) within errors of ion transmission in the drift tube. Two product ions for 2-butanone were seen at  (protonated monomer) and  (proton bound dimer), while only a protonated monomer () was observed for sulcatone. A peak at minor signal intensity is evident at  and can be attributed to octane-2,7-dione in sulcatone.^37^ Resolving powers (ratio of the drift time to the full width at half-maximum) at ion shutter pulse widths of 250 and 500 μs were 34 and 15, respectively, for the proton-bound dimer of 2-butanone. Changes in ion signal intensity when the sample is introduced to the drift tube in the UDF-mode are shown in Figure 2 as plots of ion intensity for mobility extracted ions versus elapsed time for 2-butanone (top frame) and sulcatone (bottom frame).

In the top frame for 30 ppbv of 2-butanone, ion intensities reach a maximum at 2 s for both protonated monomer and proton bound dimer, while intensity returns to baseline in ∼6 s for M_2_H^+^, and in 15 s for . Comparably behavior was observed 20 ppbv sulcatone (Figure 2 bottom frame) with a maximum response ∼3 and 10 s to baseline. Corresponding times for the octane-2,7-dione were 6 and 20 s, respectively.

Ion intensities with time can be attributed only to flow transport in the injection port and reaction region of the drift tube. In this mode of operation, the flow of drift gas and sample vapors is counter to ion flow in the electric field where ion swarm velocities are 4–7 m s^–1^. Since the inner volume of the reaction region of 23.6 cm^3^ and drift gas flow is 150 mL min^–1^, sample vapors should be vented from the reaction region in ∼9.5 s and agree with experimental findings for sulcatone. Minor differences are attributed to the uneven distribution of sample vapors mixing in the reaction region and produced by jetting of flows from the capillary (injection port) (Figure S12) and diffusion.

### Response with Ion Source in SFCV Mode

**Figure 3:**
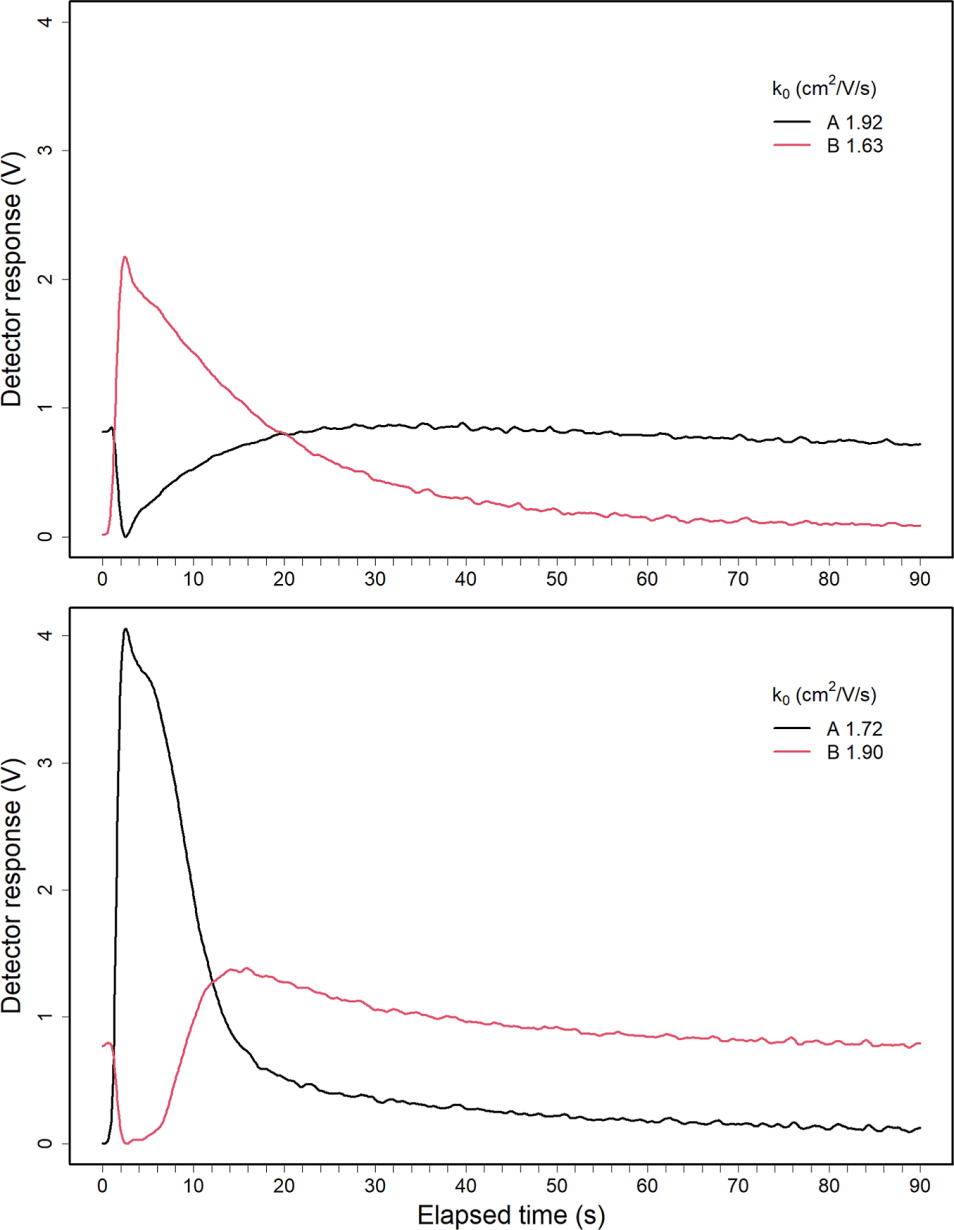
Top frame: 2-butanone sample ion peak intensities when 50 μL at 30 ppbv was injected into the reaction region in the SFCV mode. Signals are (A black,—) 2-butanone monomer and (B red,—) 2-butanone dimer. The offset of 2-butanone monomer is caused by an overlap with the reactant ion peak. Bottom frame: the sulcatone sample ion peak intensities over time when 50 μL at 20 ppbv of sample was injected to the reaction region in the SFCV mode. Signals are (A black,—) sulcatone monomer and (B red,—) octane-2,7-dione monomer. The offset seen in the octane-2,7-dione monomer peak is caused by an overlap with the reactant ion peak.

Response to samples of 2-butanone and sulcatone with the ion source in the SFCV mode is shown in Figure 3 and can be directly compared to findings in Figure 2 with identical sample amounts. In the SFCV mode, drift gas from the reaction region was diverted continuously from the reaction volume until product ion intensities reached or neared baseline response. While the product ions formed with the SFCV mode and the time to maximum response, by compound, were the same as in the UDF mode (e.g., 4 s to maximum response for 2-butanone), the time for signal to return to original conditions was nearly 8× longer, e.g., 60–70 s for the proton bound dimer of 2-butanone. Similarly, sulcatone exhibited maximum in response for the protonated monomer of ∼4 s with return to baseline in ∼80 s (Figure 3, bottom). Significantly, a difference in behavior was observed for octane-2,7-dione sulcatone impurity in both time to maximum (∼12 s vs 6 s in UDF mode) and in return to baseline (∼80 s vs 20 s). This constituted a chemical mixture and the first evidence of an expression of reaction selectivity ion formation. In the SFCV mode, the signal intensity for 2-butanone monomer at  (which overlaps the reactant ion peak) drops (Figure 3, top), while the intensity of 2-butanone dimer doubles compared to the UDF mode. This is a result of a prolonged reaction time. The response times are summarized in Table 1.

**Table 1:** Response and Clear Times for Individual Substances in UDF and SFCV Modes Measured from the Moment of Sample Introduction

	UDF mode		SFCV mode	
sample	full response (s)	clear (s)	full response (s)	clear (s)
2-butanone monomer	2	8	2	60–70
2-butanone dimer	2	15	22	60–70
sulcatone	4	10	2	80
octane-2,7-dione	6	15	12	80

**Figure 4:**
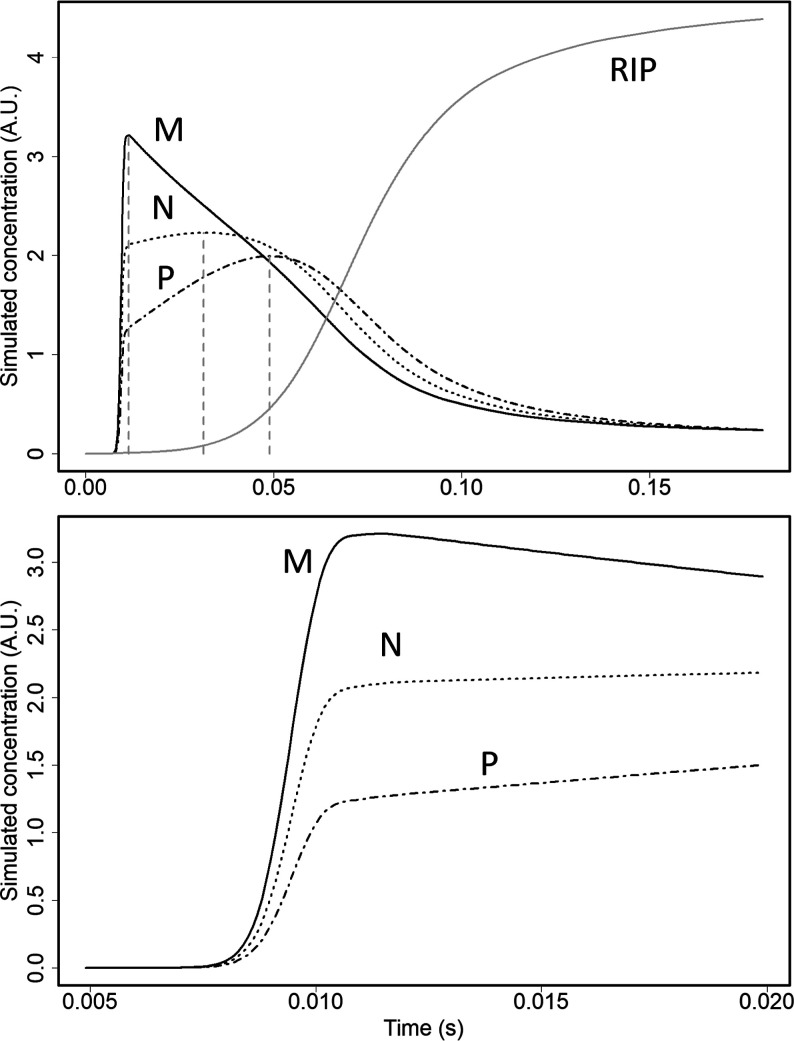
Simulated ion quantities of unimolar (1 ppbv) ternary mixture in the 2D SFCV model. The black solid (—) trace is the intensity of the chemical with the highest reaction coefficient (M), the dotted trace (·····) is the second highest (N), and the dash-dotted (---) is the lowest one (P). The lower frame shows a zoomed response between 0.005 and 0.020 s, depicting the rising edge of the product ions. The top frame shows the full temporal response of the ternary mixture. The solid gray trace (—) is the quantity of reactant ions, and vertical dashed lines show the maximums of product ion responses. The time between 0 and 0.007 s reflects the drift time from a reactant ion entrance to the “detector”. The magnitude of the time scales in computational model differ from experimental time scales as described in the chapter computational modeling.

Switching from the UDF to SFCV mode resulted in an 170% increase in signal (2.4 V, Figure 2 to 4 V, Figure 3) and this may be attributed to the extended residence times of the neutral sample vapors, understood also as the time-dependent concentration of neutral sample vapors in the reaction region (Figure 1, Section C). In the UDF mode, sample vapors are swept from the reaction region by drift gas flow, thus limiting time available for reactions with hydrated protons. Models (Figure S12) suggest that losses of sample neutrals in the SFCV mode occurs through ventilation (i.e., vapor diffusion and gas flow via capillary inlet injection) but up to 30% of sample neutrals can remain in the reaction region after 4 s in the SFCV mode, noting that estimates of rates of ventilation are speculative. The ventilation and disturbance of concentrations can also happen through the corona wind. This was not considered in the computational models.

### Models for Response with Ternary Mixture in the Reaction Region

Plots are shown in Figure 4 (top frame) for ion quantities derived from COMSOL models of reactions with the reaction region in the SFCV mode for a three component mixture, M, N, and P (see the Supporting Information, Computational Model of Confined Sample). In the bottom frame, the response is shown with 0.02 s period zoomed on the *x*-axis and shows the time of response in the millisecond range, where now the contribution from ion passage in the drift tube to the detector is evident: in the zoom of reaction time, the formation of each ion is nearly simultaneous; the appearance of ions at 0.007 s corresponds in the model to the drift time of ions through the reaction region of the simulated model (see Figure S11). It can be observed that the appearance of ions is similar, although the rate of formation is not, and the signals scale with the magnitude of rate coefficients (highest reaction rate *k*_r_ shows the largest slope). It can also be observed that the maximum ion yield also scales with the magnitude of rate coefficients. As time exceeds 0.01 s, the most neutrals for M have formed product ions and been extracted, and the ion quantity decreases. For the first 0.025 to 0.03 s, the abundance of reactant ions (in the “detector”) is nearly zero. The maximum response for each chemical differs according to the first reaction and cross-reaction rate coefficients (lower rate coefficient and longer time to maximum quantity) and is shown with cursors in Figure 4.

The simulation shows the formation of multiple product ion species as the reactant ions and neutral molecules mix within the vapor volume. The extent of product ion formation was determined by the relative magnitudes of the rate coefficients because the initial concentrations of all components were the same. The cross-reactions eqs S1–S3 and Table S4 become possible as soon as the product ions have been formed and are drawn by the electric field through the cloud of neutrals. The highest slope of compound M seen in Figure 4 can be explained by cross-reactions with product ions of P to N, from N to M and from P to M. This process is dominant during the first 0.01 s. When the concentration of neutral M decreases, the probability of direct formation of product ions for M as well as probability of cross-reactions toward M decrease as well, which is seen as a decreasing slope from about 0.01 to 0.150 s. At the same time, the cross-reactions from P to N gain importance and quantity of product ions for N increase until the concentrations of both neutrals M and N have decreased to the level where probability of product ion formation for P is the highest of all. The reactant ions are not seen in the exit boundary until the total concentration of neutrals decreases below the concentration of reactant ions.

The model supports the hypothesis of separation of ions based on kinetics when neutrals are not ventilated from the reaction space, thus leaving time for first primary reaction and then more importantly for cross-reactions and when the reaction balance is changed by removing the product ions by electric field.

### Time Resolved Response with Ternary Mixture

**Figure 5:**
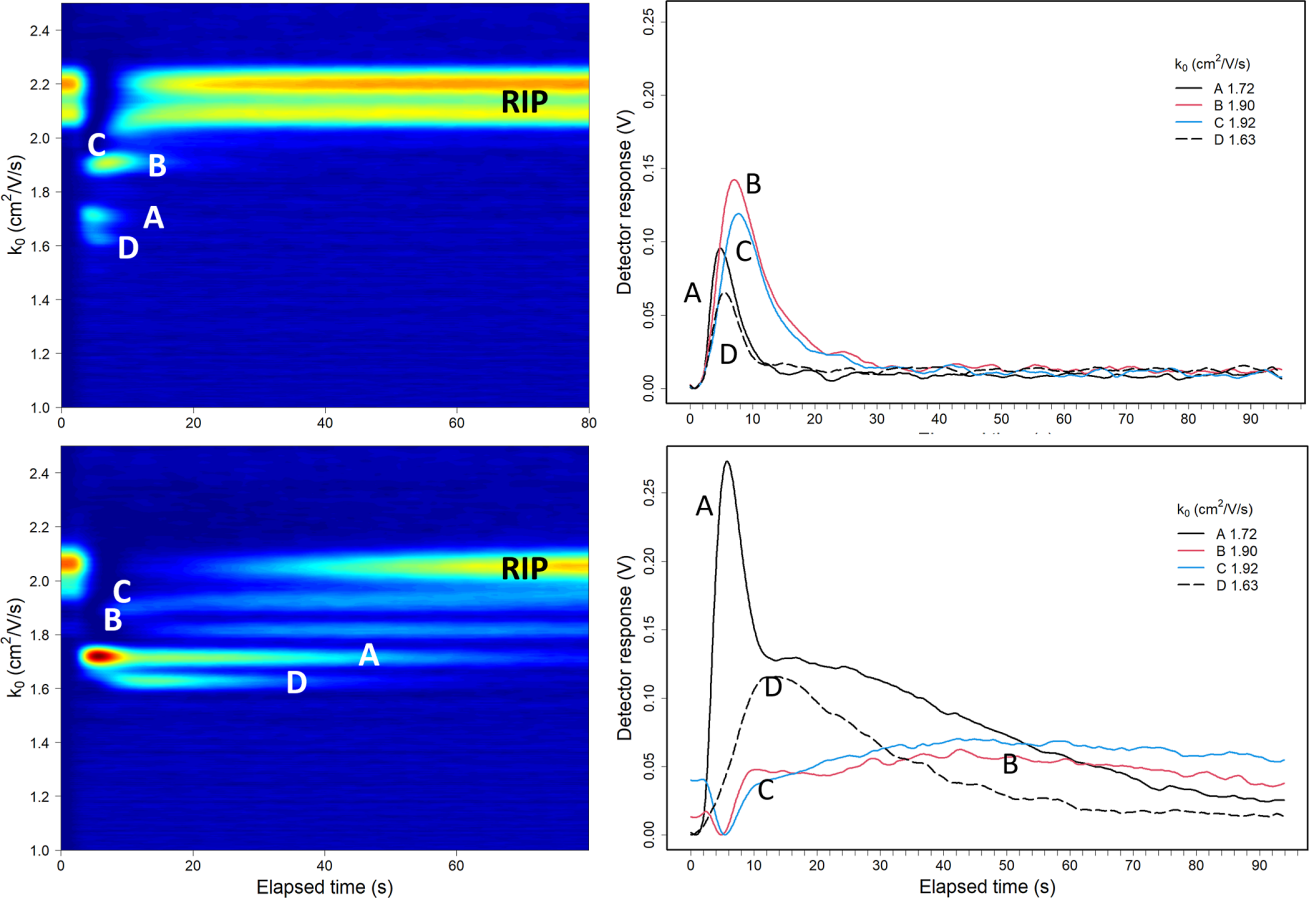
Results from analyses of a ternary mixture of sulcatone (50 ppbv), 2-butanone (80 ppbv), and octane-2,7-dione (∼1 ppbv) using UDF (top) and SFCV (bottom) modes. Ions are (A black—) sulcatone protonated monomer, (B red—) octane-2,7-dione protonated monomer, (C blue—) 2-butanone protonated monomer, and (D dashed black ---) 2-butanone proton bound dimer. Reactant ions are hydrated protons (eqs 1 and 2) and are labeled RIP. The color palette is log-scaled for enhanced visualization of peak intensities from blue (low) to red (high).

The topographic plot in Figure 5 (top frame) shows results for IMS analysis as ion signal intensity (*z*-axis), reduced mobility (*y*-axis), and elapsed time after sample injection into the drift tube (*x*-axis). Time to vent sample from the reaction region was rapid (Figure 5, top, right), as with individual constituents (Figure 2, top), with little separation in the mobility-time space (Figure 5, top left) for product ion intensities for the IMS with UDF mode (top), whereas in the SFCV mode (Figure 5, bottom), the ion signal intensities of individual constituents exhibit temporal separation. The signal intensities were only about 1/10 in Figure 5 compared to Figure 2 since the pulse width of the ion shutter was reduced to 250 from 500 μs to increase spectral resolution.

The response and clear times are compared in Table 2. The ion signal intensities of sulcatone monomer and 2-butanone proton bound dimer reached maximum intensity in 2 s, followed by octane-2,7-dione protonated monomer and 2-butanone protonated monomer in 4 s, and cleared back to base level between 14 and 30 s in the UDF mode. Responses to the same sample using the SFCV mode show temporal distribution. Signal intensity of protonated monomer of sulcatone reached maximum in 2 s, proton bound dimer of 2-butanone in 10 s, and protonated monomer of octane-2,7-dione and 2-butanone in 50–60 s. The clear down time exceeded 90 s for all compounds in the SFCV mode.

**Table 2:** Response and Clear Times for Ternary Mixture in UDF and SFCV Modes Measured from the Moment of Sample Introduction

	UDF mode		SFCV mode	
sample	full response (s)	clear (s)	full response (s)	clear (s)
2-butanone monomer	4	30	50–60	>90
2-butanone dimer	2	14	10	>90
sulcatone monomer	2	14	2	>90
octane-2,7-dione monomer	4	30	50–60	>90

Compared to the UDF mode, the SFCV mode shows larger intensities for protonated monomer of sulcatone and proton bound dimer for 2-butanone. The peak intensity was 270% for the sulcatone monomer and about 200% for the 2-butanone dimer in the SFCV mode compared to the UDF mode, and the responses in the SFCV mode also show a larger sum of intensity. For octane-2,7-dione and 2-butanone monomer, the peak intensities are lower in SFCV mode than in UDF mode, but the signals are spread over long response time.

The notable difference between the UDF and SFCV modes can be seen in signal dynamics. The responses in UDF mode can be understood with eqs 1 and 2 and combination of dilution of neutrals by drift-flow and removal of product ions by electric field: the rate of ion formation is almost equal for all compounds and the concentration of neutrals is diluted by the drift flow moving the balance from sulcatone monomer and 2-butanone dimer to octane-2.7-dione monomer and 2-butanone monomer.

The dynamics of SFCV mode can be better understood with eqs S1–S3, the differences in proton affinities, and small continuous, and thus also diluting flow from capillary injection port: as in the UDF mode, all neutrals form (primary) product ions (eq S1), but in the SFCV mode, the extended dwell time of product ions in neutral filled reaction region favors the formation of product ions with the highest PA through the cross reactions between the primary product ions (eq S3). This is seen as the most prominent response of the sulcatone monomer in Figure 5 (bottom right) followed by the 2-butanone dimer. As soon as sulcatone monomers are formed, they are also removed by the electric field and the concentration of sulcatone is diluted, allowing formation of 2-butanone dimers from 2-butanone monomers. In 10 s, the rate of formation of sulcatone monomers and 2-butanone dimers are almost balanced and sulcatone is then diluted nearly constant rate, while 2-butanone dimer and monomer responses resembles the response in Figure 3 top and response of octane-2,7-dione resembles response in Figure 3 bottom. The dilution effect of capillary flow (∼4 mL·min^–1^) is understood to be negligible at the beginning of responses, but increases over time and the venting becomes meaningful when time exceeds 300 s.

### Qualitative Study with Multicomponent Mixture

**Figure 6:**
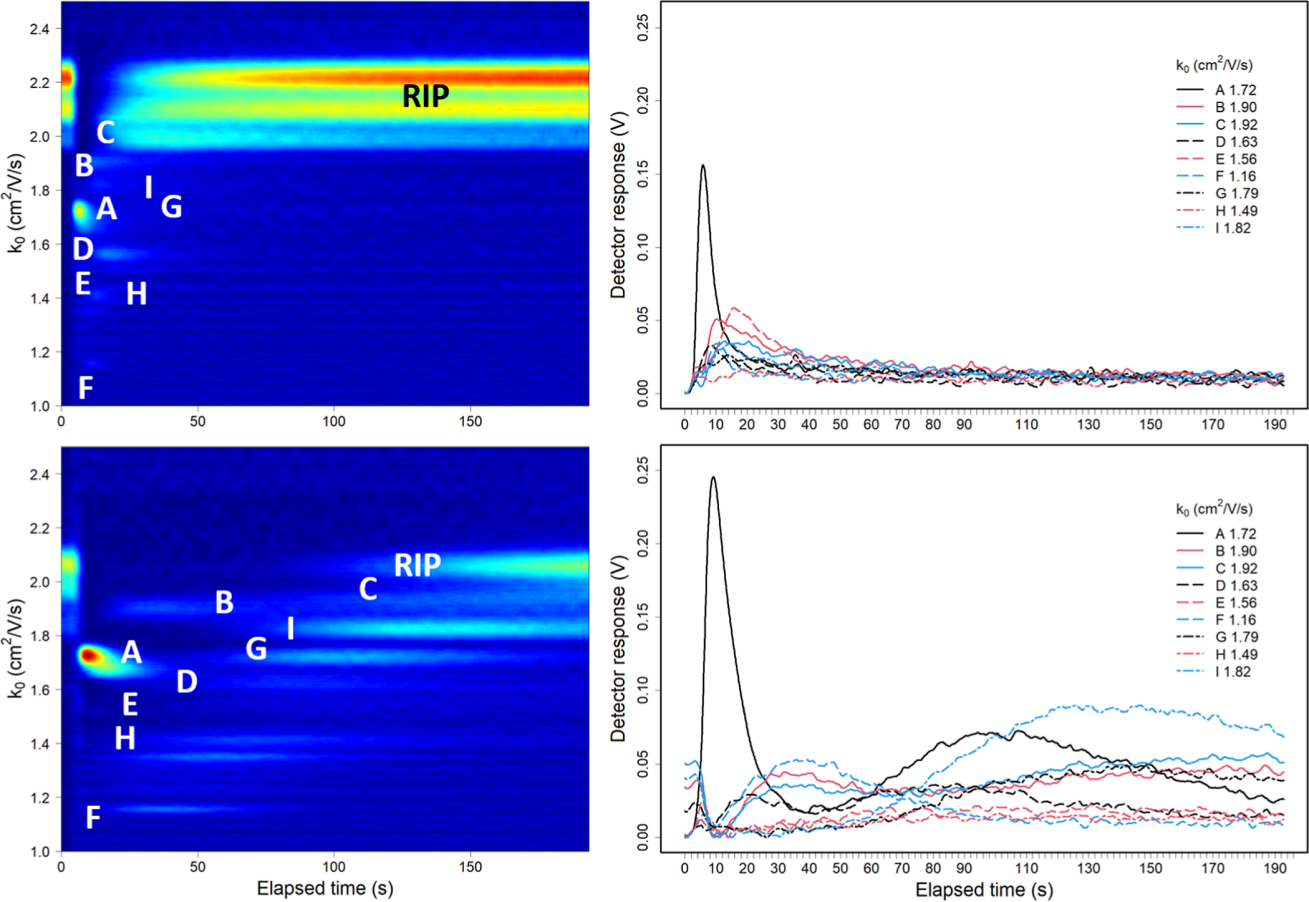
Topographic plot of reduced mobility vs elapsed time (left) and ion signal intensities vs elapsed time (right) of experiment of five-compound mixture in the UDF mode (top frames) and in the SFCV mode (bottom frames). The blue color presents the baseline, and the dark red is the highest intensity. (A black—) Sulcatone monomer, (B red—) octane-2,7-dione monomer, (C blue—) 2-butanone monomer, (D dashed black ---) 2-butanone dimer, (E dashed red ---) pentyl acetate monomer, (F dashed blue ---) pentyl acetate dimer, (G dash dotted black ---) 1-heptanol monomer, (H dashdotted red ---) 1-heptanol dimer, and (I dashdotted blue ---) isopropyl alcohol monomer. Axes are *z*-ion intensity, *y* drift time, and *x* experiment time. The concentration of each component was 25 ppbv. IMS was operated with 250 μs shutter speed for enhanced resolution. The color palette in topographic plots is log scaled for visualization.

Results from an analysis of a multicomponent mixtures are shown in Figure 6 for UDF (top) and SFCV (bottom) modes as a topographic plot of ion intensity (*z*-axis in log scale), mobility drift time (*y*-axis), and experiment time (*x*-axis). The mixture was prepared from 2-butanone, sulcatone (including octane-2,7-dione), isopropyl alcohol, 1-heptanol, and pentyl acetate, all at 25 ppbv. The separation concept using SFCV mode has been repeated with similar equipment in another laboratory and another person, although the ion mobilities as *k*_0_ are not directly comparable due to differences in operational conditions as pressure (see Supporting Information, chapter five: component mixture measured in SFCV mode at NMSU, USA and Figures S7 and S8).

The approximate response times and clear times for the data presented in Figure 6 are given in Table 3. The response in the UDF mode arises within 4 to 16 s for all ions, resembling the behavior seen in Figure 5 top. Ion peaks can be observed at sulcatone monomer , 2-butanone monomer , sulcatone impurity monomer , 2-butanone dimer , pentyl acetate monomer , and 1-heptanol monomer . The abundance of ions in UDF-mode is low, and the responses are cleared in 70 s. In contrast to other compounds, the response to sulcatone is fast and dominant.

**Table 3:** Approximate Full Response and Clear Times for Multi-Component Mixture in UDF and SFCV Modes Measured from the Moment of Sample Introduction

			UDF		SFCV	
chemical	*k*_0_	label	full resp. (s)	clear (s)	full resp. (s)	clear (s)
sulcatone monomer	1.72	A	4	>70	10	>190
octane-2,7-dione	1.90	B	8	>70	28	>190
2-butanone monomer	1.92	C	12	>70	30	>190
2-butanone dimer	1.60	D	6	>70	16	>190
pentyl acetate monomer	1.56	E	14	>70	90	>190
pentyl acetate dimer	1.16	F	10	>70	32	>190
1-heptanol monomer	1.79	G	12	>70	140	>190
1-heptanol dimer	1.49	H	16	>70	60	>190
isopropyl alcohol monomer	1.82	I	10	>70	120	>190

In the SFCV mode, the maximum number of ions other than sulcatone monomer have been shifted. The signal intensity of 2-butanone dimer has the first maximum at 10 s and the second one at 90 s after the start of exposure. Pentyl acetate dimer (), 2-butanone monomer (), and octane-2,7-dione () reach maximum about 28–30 s after the start and isopropyl alcohol monomer reaches maximum about 120 s from the beginning.

The SFCV mode exhibits dynamical behavior (eqs S4–S6) of ion signal intensities, which demonstrate the charge competition between the compounds in the confined volume in the presence of continuous feed of reactant ions from the ion source and the removal of product ions by electric field. In the UDF-mode, the charges from reactant ions are shared between all compounds, but the time for cross-reactions is short because the sample is pushed toward the ionization region by the drift flow and sample is diluted, while ions are extracted by electric field. In the SFCV mode, the most reactive compound, sulcatone, is washed first from the reaction region. The reactions, in this mixture, are favorable next for pentyl acetate, 2-butanone, and sulcatone impurity, matching the order of proton affinities. The delayed ion signal intensities for other compounds are a result of their position in cross-reaction competition.

The charge sharing in UDF mode IMS limits detection of components in the mixture through the overlap of peaks from two or more chemicals. In contrast, the ionization of VOCs in a mixture with the SFCV mode shows a time-resolved separation of ion peaks. The product ion quantity depends on the quantity of reactant ions (limited by the ionization source), rate coefficients between neutral and reactant ions, concentration of neutrals, and rate coefficients of cross-reactions between the product ions and other neutrals, and thus, the temporal order of ion signal intensities cannot be predicted in the case of an unknown sample. However, the benefits of temporal separation can be seen as increased selectivity via simplified peak separation and increased sensitivity via increased intensity of single compounds. It is notable that the experiment presented in this paper differs from an ideal one in several manners:

1. In the ideal construction, the sample is evenly distributed over the reaction region. In the experiment, the sample is introduced as a manual controlled pulse from a syringe using GC-inlet, leading to an unknown distribution of the sample in the reaction region. Also, sample concentration distribution changes as a result of ionization when ions annihilate in the grid and are turned back to neutrals near the grid.
2. In the ideal construction, the convection of the sample is zero. In the experiment, sample is the introduced with GC-inlet and continuous carrier gas. Although the volume rate of the carrier is low, the convection of the sample can result in dynamic variations of sample concentration within the reaction region and sample dilution over time. Furthermore, the sample introduction by manual injection introduces variations in intensity of responses. Also, the corona source creates corona wind,^46^ which further distorts the sample distribution.
3. In the ideal construction, there are no surface reactions. Although the experiment is used in elevated temperatures to minimize adsorption to desorption from the surfaces, there is a risk that the results are affected of such reactions.

Despite the limitations in our experiment, the temporal separation of substances, demonstrated in Figures 5 and 6, improves the capability for the characterization of constituents in a mixture via separated ion mobilities.

## Conclusions

The time-resolved behavior of ion signal intensities when a sample volume is retained in a reaction region for more than 20 s is consistent with a separation of vapors based on ionization reactivity. Findings were consistent with models from finite element methods where the response was governed by reaction volume geometry, mass transport of ions, and cross-reactions among constituent vapors and corresponding protonated species. This additional selectivity of response provides another dimension to IMS while maintaining the simplicity of drift tube design and operation. Although the concept of the SFCV reaction region has been introduced in this work, the process of separation by reaction chemistry has also been termed as GID for wider applications. Limitations in GID are currently attributed to possibly uneven sample distribution in the reaction region during sample introduction and optimization of sample residence, reactant ion-sample vapor mixing, and reaction region dimensions.

## Supporting Information Available

Description of the test instrument, API reactions behind simulation discussed in chapter models for response with the ternary mixture in the reaction region, method of flow control in SFCV ion mobility spectrometer, single component spectra of sulcatone and 2-butanone samples, spectra of binary mixtures in UDF- and SFCV-modes, five-component mixture measured in the SFCV mode at NMSU, USA, computational modeling, testing the separation hypothesis with simulation, computational model of confined sample, and study of sample injection via GC-capillary split valve into a SFCV (PDF)

## Notes

All authors declare that the manuscript has been written solely by authors and no Artificial Intelligence (AI) text generation has been used.
