# Supplementary material for: Time-Resolved Ion Mobility Spectrometry with a Stop Flow Confined Volume Reaction Region
Source: Anal Chem. 2024 Jun 10;96(25):10182–92. doi: 10.1021/acs.analchem.4c00434 (PMC11209659; doi:10.1021/acs.analchem.4c00434)
Supplement: Supplementary file 1 — ac4c00434_si_001.pdf [file ac4c00434_si_001.pdf]

Supporting Information

TIME-RESOLVED ION MOBILITY SPECTROMETRY WITH A STOP FLOW CONFINED VOLUME REACTION REGION

Osmo Anttalainen,[*,†] Markus Karjalainen,[†] Elie Lattouf,[†], Oliver Hecht,[‡] Paula Vanninen,[†] Hanna Hakulinen,[†] Tapio Kotiaho,[¶,‖] Charles Thomas,[§] Gary Eiceman[†,⊥]

[†] *VERIFIN, Finnish institute for Verification of the Chemical Weapons Convention, Department of Chemistry, University of Helsinki, FI-00014, Helsinki, Finland*

[‡] *Airsense Analytics GmbH, Hagenower Straße 73, 19061 Schwerin, Germany*

[¶] *Drug Research Program and Division of Pharmaceutical Chemistry and Technology, Faculty of Pharmacy, P.O. Box 56, FI-00014 University of Helsinki, Finland*

[§] *Department of Chemistry, Loughborough University, Leicestershire, LE11 3TU, UK*

[‖] *Department of Chemistry, Faculty of Science, P.O.Box 55, FIN-00014, University of Helsinki, Helsinki, Finland*

[⊥] *New Mexico State University, 1175 N Horseshoe Dr. Las Cruces, NM 88003, USA*

E-mail: osmo.anttlainen@helsinki.fi

Contents

	Page
Description of the test instrument	S-2
API reactions	S-5
Method of flow control in Stop Flow Confined Volume Ion Mobility Spectrometer	S-7
Single component spectra of sulcatone and 2-butanone samples	S-9
Spectra of binary mixtures in UDF- and SFCV-modes	S-11
Five component mixture measured in SFCV mode at NMSU, USA	S-12
Computational modelling	S-15
Testing the separation hypothesis with simulation	S-15
Computational model of confined sample	S-19
Study of sample injection via GC-capillary split valve into a Stop Flow Confined Volume	S-20

Description of the test instrument

Configuration of Stop Flow Confined Volume drift tube (Figure 1) ring distances, resistance values in resistive voltage divider, ring potentials and an electric field between the rings are shown in the Table S2.

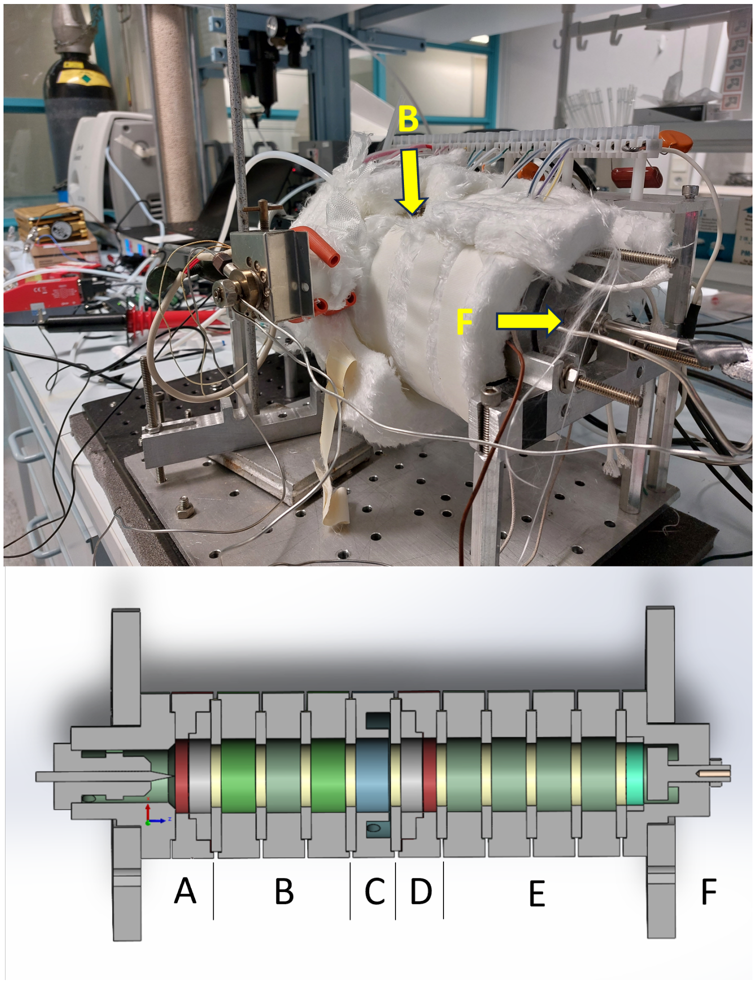

Figure S1: Top: Modified drift tube with thermal insulator interfaced with GC-inlet port. Bottom: Cut-off view of mechanical design of ion mobility spectrometer with flow control interface between extended reaction and drift regions. A: corona ionization and drift gas exit in UDF-mode, B: reaction region and sample injection, C: drift gas exit in SFCV-mode, D: ion shutter, E: drift region, F ion detector.

Table S1: SFCV drift IMS configuration. Distances are relative from ring to ring. The first distance is measured from the inner surface of the supporting structure.

Distance mm	Resistor $M\Omega$	Potential V	Field (E) $V \cdot cm^{-1}$
grid		4000	
9	2.07		326
source ring 1		3706	
13	2.98		325
source ring 2		3284	
13	2.98		325
source ring 3		2861	
9	2.07		326
gas exit		2567	
13	2.98		325
gas entrance		2145	
6	1.38		326
Ion shutter ref		1949	
4	0.92		326
Drift ring 1		1818	
13	2.98		325
Drift ring 2		1396	
13	2.98		325
Drift ring 3		973	
13	2.98		325
Drift ring 4		550	
13	2.98		325
Aperture grid		128	
3	0.9		426
ground		0	

API reactions

Atmospheric pressure ionization is a complex chain of reactions resulting to hydrated protons, called here reactant ions ($H^+(H_2O)_n$ or R^+).[35] With reactant ions API of a vapor mixture can be complex and comprised of initial formation of ions which is followed in time by secondary (or cross) reactions in a vapor cloud of substances in air. Some cross-reactions will be more favored than others based on ionization properties of individual substances. A descriptive example of possible reactions and their cross-reaction paths in case of two substances are presented in Table S2. It is notable, that primary ionization of any neutral is probable as long as reactant ions are present. Also, the cross-reactions do not become possible until at least one kind of product ion exist.

Table S2: API reactions.

**Primary ionization**	**Cross-reactions**
$N_1 + R^+ \xrightarrow{k_{N1}} N_1R^+$	$N_1R^+ + N_2 \xrightarrow{k_{N_1N_2}} N_2R^+ + N_1$
$N_2 + R^+ \xrightarrow{k_{N2}} N_2R^+$	$N_2R^+ + N_1 \xrightarrow{k_{N_2N_1}} N_1R^+ + N_2$

With the generig loss terms, simplifying notation, and considering the tiny volume the reactions in Table S2 can be described in more generic way and in dynamic form as:

$$\frac{d[R^+]}{dt} = S_{R^+} - \sum k_{N_i}[N_i][R^+] - L_{R^+} \tag{S1}$$

$$\frac{d[N_i]}{dt} = -k_{N_i}[N_i][R^+] - \sum_{j \neq i} k_{N_jN_i}[N_jR^+][N_i] \tag{S2}$$

$$\frac{d[N_iR^+]}{dt} = k_{N_i}[N_i][R^+] + \sum_{j \neq i} k_{N_jN_i}[N_jR^+][N_i] - \sum_{j \neq i} k_{N_iN_j}[N_iR^+][N_j] - L_{N_iR^+} \tag{S3}$$

Equation S1 describe the generation S_{R^+} and loss L_{R^+} of reactant ions via reactions with neutrals, ion extraction, or for example losses with unintended convection, Equation S2 reduction of quantity of some neutral molecules via direct reactions of reactant ions or cross-reactions with some product ions, and Equation S3 dynamic development of product ions

via reactions of neutrals and reactant ions and via cross-reactions to and from the said product ions, and the losses $L_{N_iR^+}$. Equations S1 to S3 can be applied to FEM-models where source and loss terms are given as initial values and boundary conditions, and where set of equations are linked to simulated coordinates. Because the quantity of reactant ions R^+ can be considered given, the kinetics is not defined only by the reaction coefficients k_{N_i} but rather by the product of the reaction coefficient and concentration ($k_{N_i}[N_i]$). This means that the order of ionized substances in the temporal separation can not be predicted until values of both rate coefficients and neutral concentrations are known. Furthermore, the duration of temporal separation overall relies on the total concentration of neutrals and occurs most rapidly at low concentrations.

Method of flow control in Stop Flow Confined Volume Ion Mobility Spectrometer

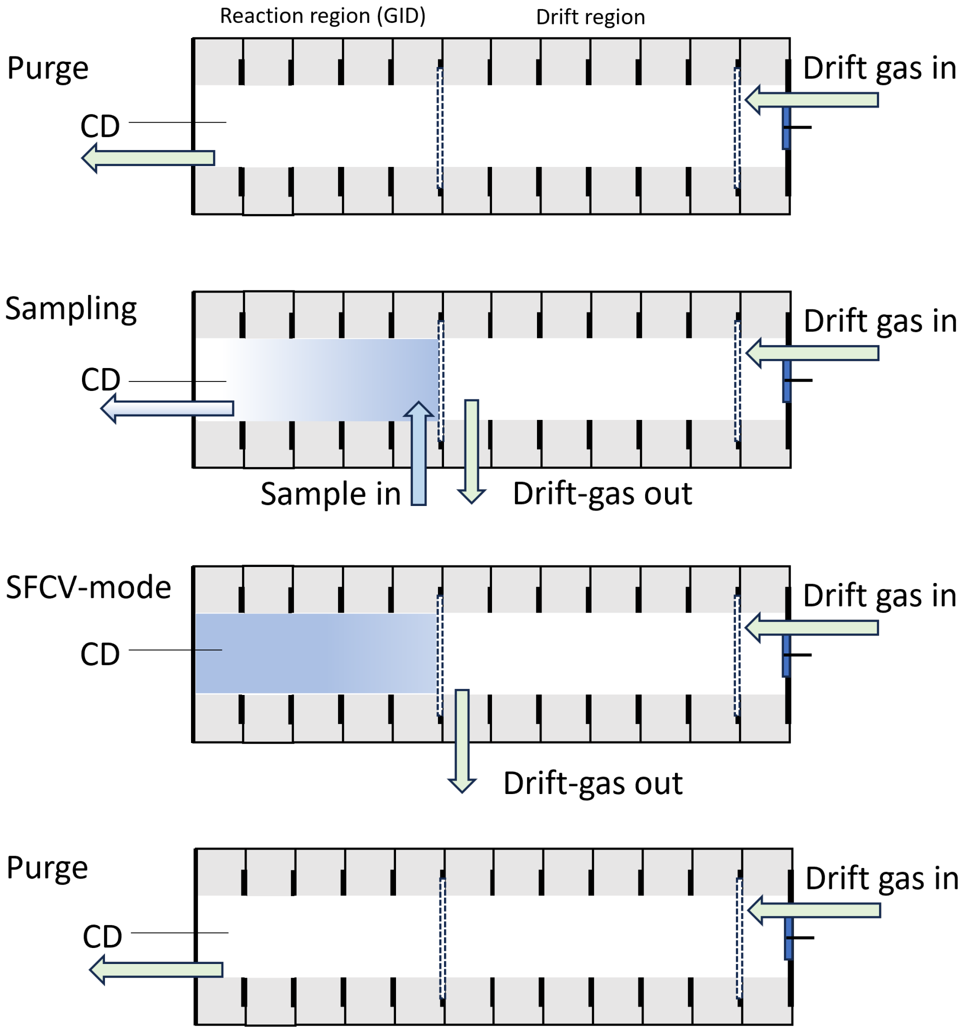

Figure S2: Method of flow control.

In common drfit-tubes the sample is introduced near ionization region and flushed away with the uni-directional drift-flow. In the Stop Flow Confined Volume Ion Mobility Spectrometer, the reaction region volume is enlargened compared to conventional drift-tubes and sample is introduced to the confined volume and ionization occurs in batch-type manner. In this process, the temporal equilibrium or dynamic balance of formed ions depends on concentration of reactant ions, concentration of neutrals, concentration of formed ions, rate coefficients and

proton affinities (positive ions) of the mixture components. The ions are removed from the confined volume in regular manner, which changes the conditions in the confined volume. The extracted portion represent ions with the highest formation probability at the extraction moment and can be observed as a temporal separation of the the mixture components. This process is called Gas Ion Distillation, GID.

Figure S2 presents the method of Flow control. In the first stage, the entire system is cleaned with purified flow through the drift region and reaction region, called as GID volume, and Corona discharge (CD). At the second stage, the drift flow is driven out from the flow drift region and sample is feeded into GID. At the third stage, sample feed is stopped, leaving the GID volume in the Stop Flow condition and reactions take place in the GID volume. Ions are gated to the drift region and their signal intensity is measured. Once the predefined residence time has passed, the process starts over again with purge stage.

Single component spectra of sulcatone and 2-butanone samples

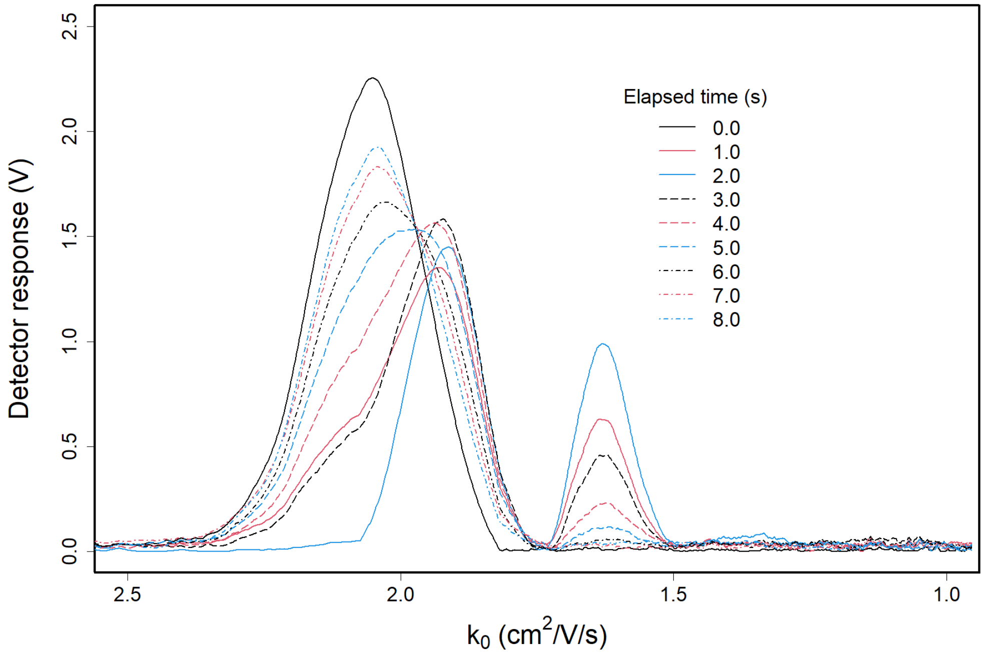

Figure S3: Spectra in one second interval of 2-butanone (30 $ppbv$) referred to elapsed time 0. Peak at 2.1 $\frac{cm^2}{V \cdot s}$ is reactant ion, peak at 1.92 $\frac{cm^2}{V \cdot s}$ 2-butanone monomer, and peak at 1.64 $\frac{cm^2}{V \cdot s}$ butanone dimer. Shutter was operated at 500 μs.

Figure S3 presents the response of IMS when 2-butanone was injected into the reaction region. The control (reactant ions) is presented at t=0 s. At t=1s the sample has filled the reaction region, and both monomers and dimers has been formed. At t=2 s, both monomer and dimer peaks reach maxima and reactant ion peak is not detected. At t=3 s, the sample has diluted in the reaction region, monomer and dimer signals have reduced from the maxima and reactant ions can be observed again. This process continues until t=8 s, where no dimer peak can be observed, and monomer and reactant peaks overlap without any separation.

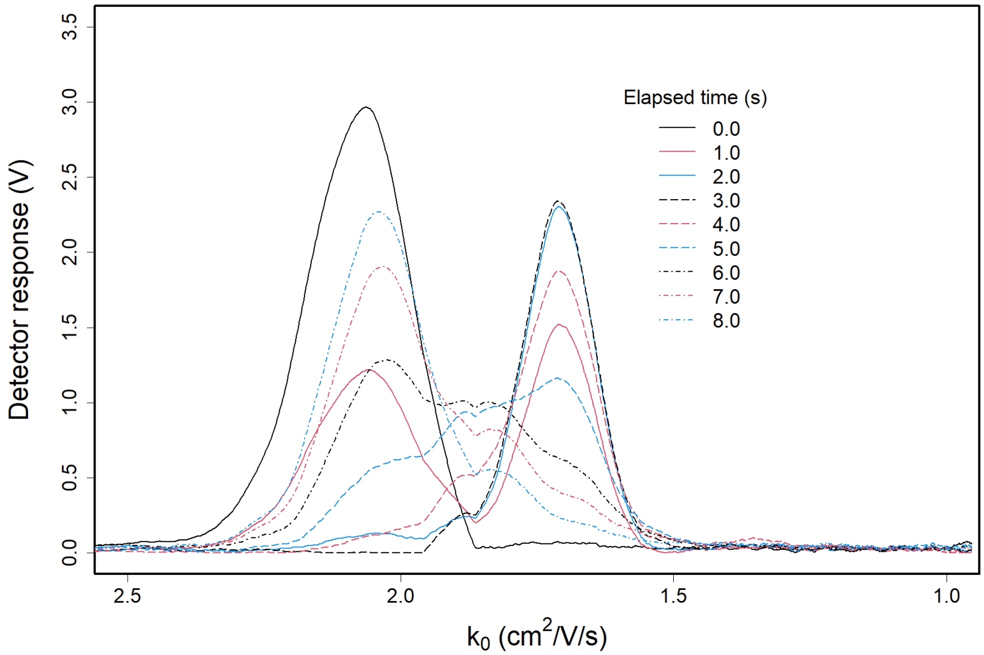

Figure S4: Spectra in one second interval of Sulcatone (20 *ppbv*) referred to elapsed time 0. Peak at 2.1 $\frac{cm^2}{V \cdot s}$ is reactant ions and peak at 1.72 $\frac{cm^2}{V \cdot s}$ is sulcatone monomer. Intensity of octane-2-7-dione (sulcatone impurity) monomer at 1.90 $\frac{cm^2}{V \cdot s}$ is not peak separated. Shutter was operated at 500 μs.

Figure S4 presents the response of IMS when sulcatone was injected into the reaction region. The control (reactant ions) is presented at t=0 s. At t=1s, sulcatone sample has filled the reaction region, but has not yet reached the maximum. At t=2 s, and t=3s, the sulcatone peak has reached maximum. At t=4 s and t=5 s, the octane-2,7-dione (impurity in sulcatone) response is seen, and the return towards the baseline can be observed when t=8 s.

Spectra of binary mixtures in UDF- and SFCV-modes

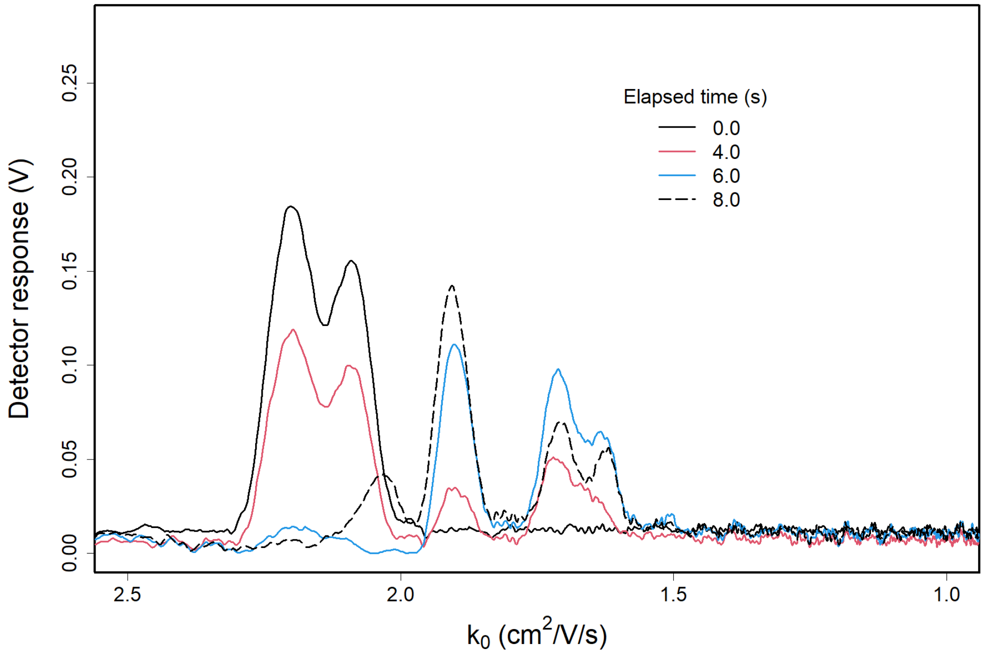

Figure S5: Selected spectra at selected moments referred to elapsed time 0 from Figure 5 top panel (UDF-mode), with 20 *ppbv* of 2-butanone and 50 *ppbv* of sulcatone sample. Peak at 2.1 $\frac{cm^2}{V \cdot s}$ is reactant ions, peak at 2.2 $\frac{cm^2}{V \cdot s}$ is assumed to be ammonia and is unresolved when shutter is operated at 500 μs, unresolved peaks at 1.93 $\frac{cm^2}{V \cdot s}$ and 1.88 $\frac{cm^2}{V \cdot s}$ are 2-butanone monomer and sulcatone impurity monomer respectively, and unresolved peaks at 1.72 $\frac{cm^2}{V \cdot s}$ and 1.64 $\frac{cm^2}{V \cdot s}$ sulcatone monomer and 2-butanone dimer respectively. Shutter was operated at 250 μs.

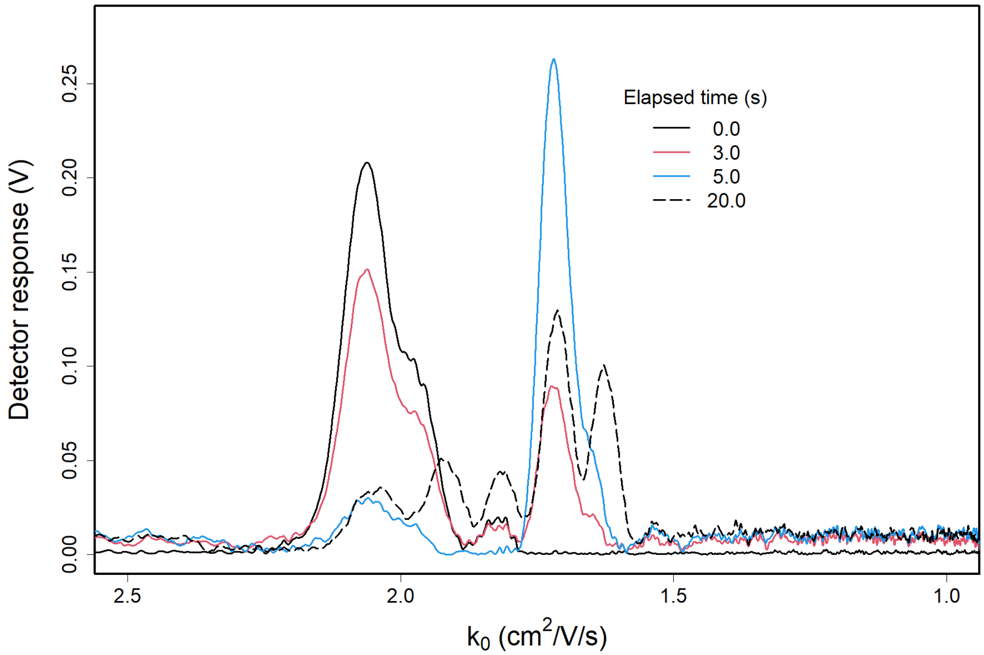

Figure S6: Selected spectra at selected moments referred to elapsed time 0 from Figure 5 bottom panel (SFCV-mode), with 20 *ppbv* of 2-butanone and 50 *ppbv* of sulcatone sample. Peak at 2.1 $\frac{cm^2}{V \cdot s}$ is reactant ions, unresolved peaks at 1.93 $\frac{cm^2}{V \cdot s}$ and 1.88 $\frac{cm^2}{V \cdot s}$ are 2-butanone monomer and sulcatone impurity respectively, and peaks at 1.72 $\frac{cm^2}{V \cdot s}$ and 1.64 $\frac{cm^2}{V \cdot s}$ sulcatone monomer and 2-butanone dimer respectively. The shutter was operated at 250 μs.

Five component mixture measured in SFCV mode at NMSU, USA

Five component mixture was measured with an equivalent instrument as used for ternary and multi-mixture experiments. Measurements were performed at NMSU Department of Chemistry and Biochemistry, Las Cruces, USA by Oliver Hecht and Gary Eiceman.

Instrumentation

GID enhanced IMS was used. IMS drift voltage 6 kV. Corona needle at 9 kV. Sample was introduced over the side port into the GID region via a gas tight syringe. The drift tube and drift gas were heated to 50 $°C$.

Sample preparation

An equal molar mixture was produced. Therefore, 2 μL of 2-butanone (Sigma-Aldrich with $\geq$ 99% purity), 3.3 μL of sulcatone (Sigma-Aldrich with 99% purity), 3.3 μL of pentyl acetate (Sigma-Aldrich with 99% purity), 1.17 μL of 1-Heptanol (Sigma-Aldrich with 98% purity) and 1.7 μL of 2-propanol (Sigma-Aldrich with 99.9% purity) were vaporized in a heated 1000 mL glass flask. After 10 min 5.68 mL were taken out from this flask and were diluted into a heated 500 ml glass flask. After another 10 min 100 μL of the diluted mixture were given into the GID volume (15.2 mL) using a gas tight syringe (Hamilton gas tight 1001) so in the GID volume a sample concentration of 50 $ppbv$ is reached for each chemical.

Measurements

The sample was injected 5 times and 15 ms long mobility spectra were averaged over 5 spectra, but ions were not correlated for single compounds. The average progression of the peak heights of each peak after an injection are plotted in Figure S8. The labels in the Figures S7 and S8 are to link corresponding signals in topographical and ion intensity signals.

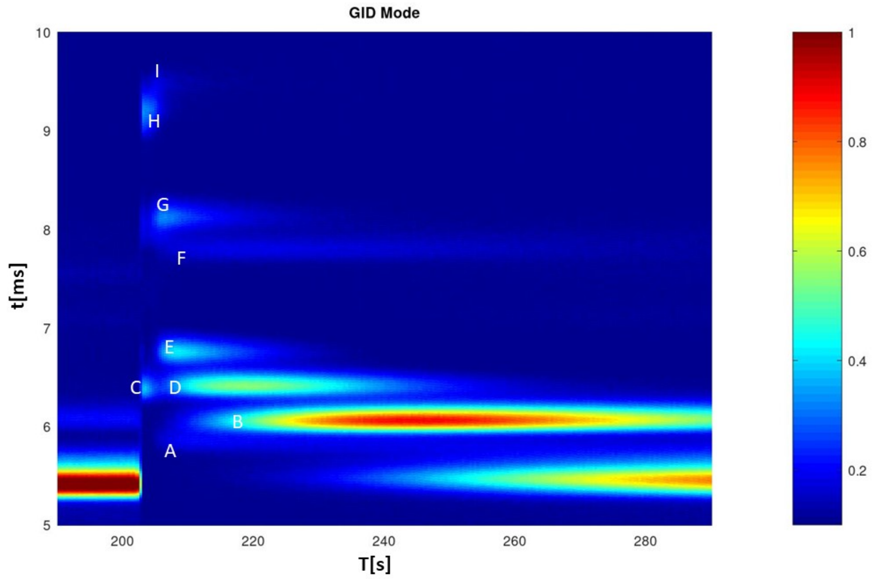

Figure S7: Topographical plot of 5-component mixture averaged over 5 sample repetitions vs elapsed time. Colors show the relative signal intensity of ions.

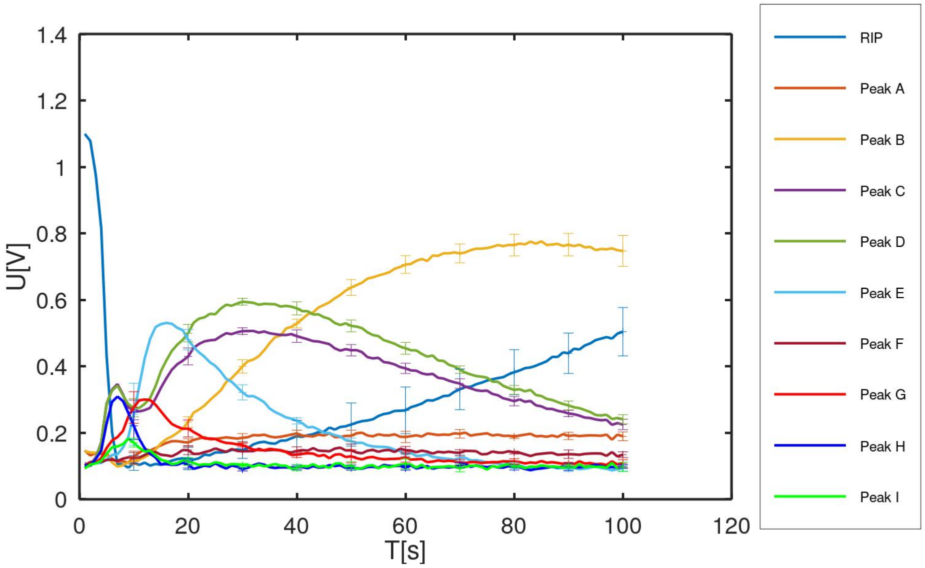

Figure S8: Ion signal intensities of averaged intensities of selected ion mobilities as detector response vs elapsed time of 5 component mixture. The error bars show 4-sigma deviation between the averaged samples. The legend letters refer to Figure S7.

Computational modelling

Reaction-based simulation predicts that separation of equal concentrations of neutrals M and N is a possible if there is difference in reaction rate coefficients.[37] Plain reaction-based simulation does not account for effects from simulation space geometry, dynamic or spatial effects of sample introduction, gas flow in the reaction region, or transportation of ions in the electric field.

Testing the separation hypothesis with simulation

To study the realism of separation, while still idealized environment, a computational test model of reaction space followed by small drift-tube was simulated with Comsol multiphysics.

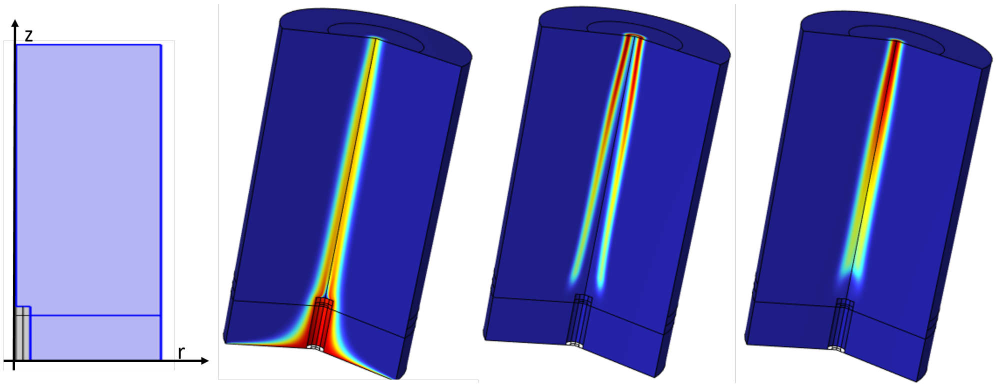

Figure S9: Hypothetical arrangement of GID as simulation model geometry to study effects of geometry and construction, and simulated concentration of reactant ions while counterflow was kept zero. From left to right: axisymmetric section of the model, R^+, product ions MR^+ and product ions NR^+ at 60 ms when drift-field potential was set to 500 V, and sample concentration of M and N was 100 ppb. The sample was injected as 0.1 ms pulse at flow rate of 5 $mL \cdot min^{-1}$.

The Figure S9 presents arrangement of the model and simulated concentrations of reactant ions R^+, product ions MR^+, and NR^+ modelled as a cylinder symmetric tube. The transportation of neutrals was based on the convection simulated with laminar-flow module of Comsol Multiphysics. The transportation of ions was simulated with the Transport of Diluted Species module using steady state solutions from the electrostatics and laminar flow modules for transportation by electric migration and convection as fixed field values (assum-

ing that ion movement does not infer flow or field). The transportation included diffusion, which was described via parameters of electrical mobility. The concentrations of product ions were measured as an average concentration over time at the topmost boundary of the model (which was set to the ground potential). In the Figure S9 the tube length is 35 mm and the radius is 20 mm. The sample was introduced as an injection of 0.1 ms long pulse at a flow rate of 5 $mL \cdot min^{-1}$ into the central axis from a 0.5 mm diameter tube surrounded by the shield flow of 10 $mL \cdot min^{-1}$ from 1 mm diameter coaxial tube. The electric field was established by setting the bottom and first 5 mm of the outer wall of the tube to a potential of 300 V and the rest of the wall to the linearly decreasing potential, reaching zero at the end of the tube. The constant concentration of 1×10^{11} cm^{-3} of positive reactant ions were placed on the bottom of the drift tube, from where they were deflected under the effects of the electric field and flow through the tube. The chemical reactions between the neutrals and reactant ions were allowed through the tube, as presented in Equations S4 to S6.

$$R^{+} + M \rightarrow MR^{+} \tag{S4}$$

$$R^{+} + N \rightarrow NR^{+} \tag{S5}$$

$$MR^{+} + N \leftrightarrow NR^{+} + M \tag{S6}$$

Here, R^{+} represents hydrated protons, M is neutral, the MR^{+} protonated monomer of M, N is another neutral, and NR^{+} is the protonated monomer of N. The cross-reaction is described with a two-directional reaction using the parameter k_{NM} for forward and reverse reactions.

To test the separation hypothesis with binary mixture, the rate coefficient associated to data in reference[37] $k_{rN} = 1.39 \times 10^{-9} cm^{3} \cdot s^{-1}$ and $k_{rM} = xk_{rN}$ for M and N were used in the simulation (model in Figure S9) and the multiplier x was varied between 1 and 50 in

Table S3: Comsol parameters used in simulation models.

Parameter	Value	Description
x	1	Multiplier in rate coefficient simulation
k_M	$x \times 1.11 \times 10^{-9}\ cm^3 \cdot s^{-1}$	Rate coefficient for $R^+ + M$ reaction
k_N	$9.97 \times 10^{-10}\ cm^3 \cdot s^{-1}$	Rate coefficient for $R^+ + N$ reaction
k_NM	$1.39 \times 10^{-12}\ cm^3 \cdot s^{-1}$	Cross reaction coefficient
k0_RIP	$2.4\ cm^2 \cdot V^{-1} \cdot s^{-1}$	Electrical mobility of R^+
k0_M	$1.8\ cm^2 \cdot V^{-1} \cdot s^{-1}$	Electrical mobility of MR^+
k0_N	$1.7\ cm^2 \cdot V^{-1} \cdot s^{-1}$	Electrical mobility of NR^+
C0_RIP	$1 \times 10^{11}\ cm^{-3}$	R^+ source concentration
C0_M	1 *ppbv*	Sample concentration of M
C0_N	1 *ppbv*	Sample concentration of N
V_drift	300 *V*	Electrical potential to establish drift field

four scenarios. The ion abundances measured in the "detector" of the simulated system are presented in Figure S10.

When the coefficients k_{rM} and k_{rN} are near equal ($x = 1$), the reaction rate is close to equal and takes equal long time. The slight difference in response is related to difference in mobilities between MR^+ ($1.8\ \frac{cm^2}{V \cdot s}$) and NR^+ ($1.7\ \frac{cm^2}{V \cdot s}$). When coefficient k_{rM} gets larger, the quantity of product ion MR^+ increases fast and the quantity of neutrals M reduces fast, while quantity of NR^+ follows the same pattern as before. The system here is not limited by quantity of reactant ions, and thus increasing reaction rate coefficient increases the formation of product ions MR^+.

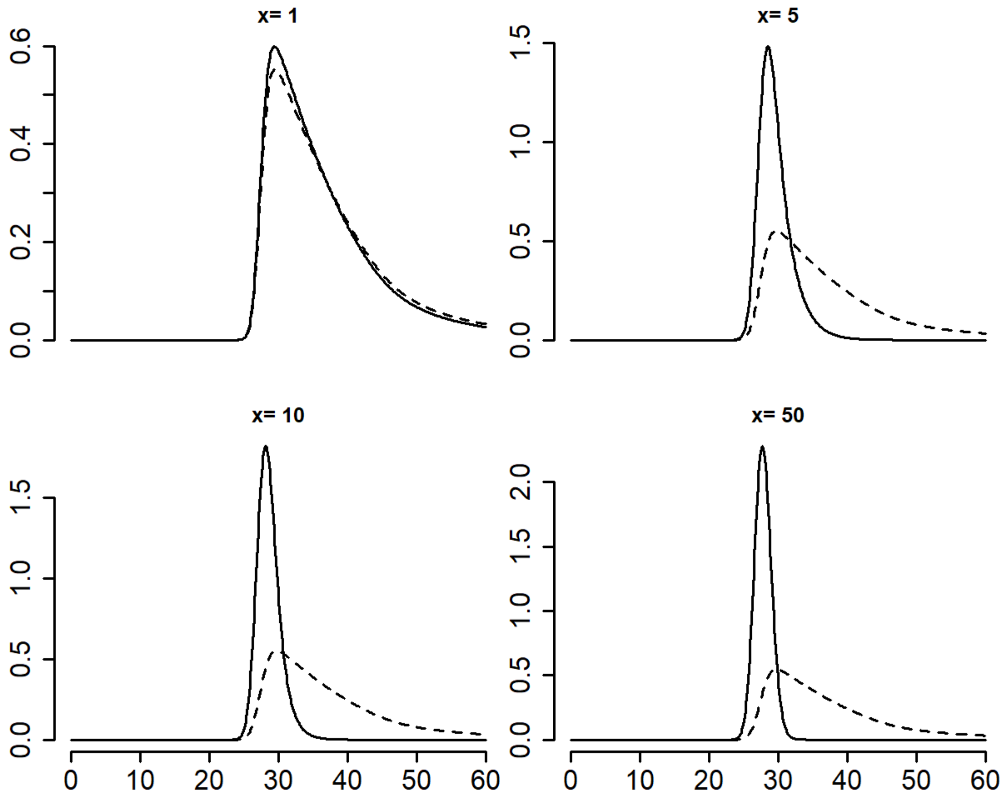

Figure S10: Simulated ion quantities of product ions of MR^{+} (solid —) and NR^{+} (dashed - - -) when k_{rM} was varied with multiplier $x = 1, 5, 10, 50$.

The concentration of both M and N was equal, and thus the probability of ionization depends only on the reaction rate coefficient. The simulation predicts that at least equimolar binary mixture can be separated in time, if the residence time of reactant ions with the sample is long enough and there is a difference in their rate coefficients. In generic case, the separation is effective only when the ratio $\frac{k_{rM}[M]}{k_{rN}[N]} \neq 1$.

Computational model of confined sample

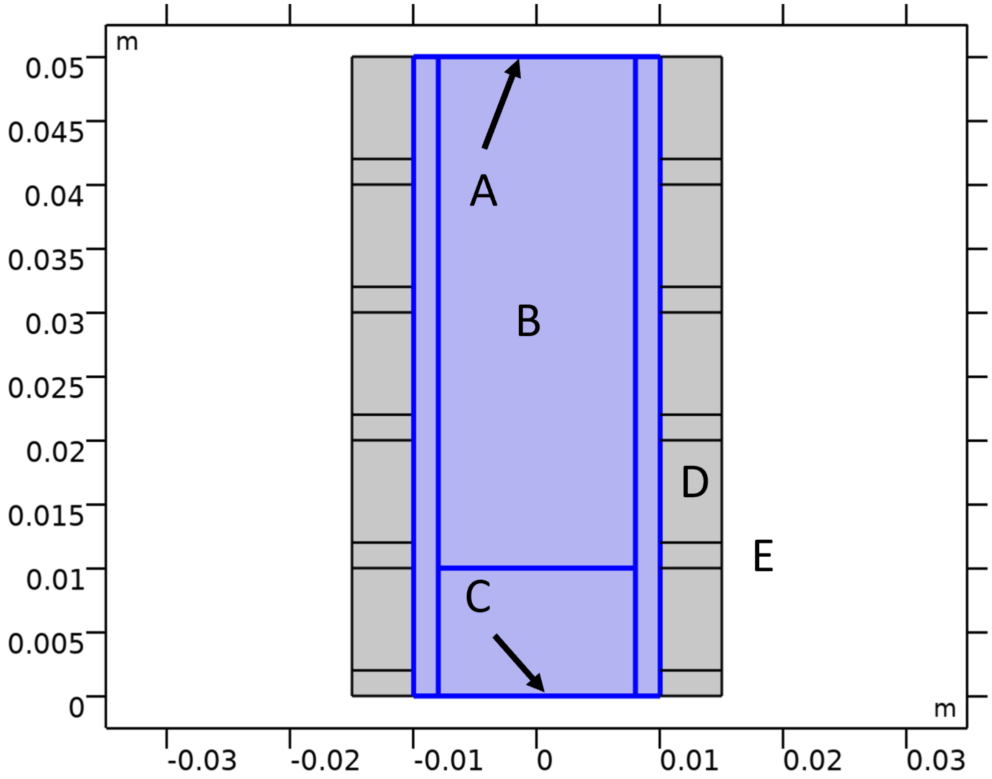

Figure S11: Confined volume sample region model simulation geometry. (A) Reactant ion entrance, (B) reaction region (all blue area), (C) ion exit / detector, (D) insulator, and (E) drift ring with electric potential. Internal lines within the blue area were used to control meshing and have no physical meanings.

The data presented in Figure 4 presents a solution from 2D Comsol Multiphysics simulation of a confined sample region presented in Figure S11. Simulation included convection (drift-flow), ion migration by electric field, diffusion, chemical reactions between neutrals and reactant ions, and also cross-reactions between the product ions. The drift flow was introduced from top to bottom (Figure S11), and reactant ions were introduced as continuous flux from the bottom. The sample mixture was introduced uniformly over the sample region. Flow and electric field were solved separately and used as stationary fields in transportation simulation. To scale the reactions into a computationally reasonable simulation period, the abundance of reactants were presented in excess, and therefore the resulting response times are only a fraction of real life response times. The total length of ion transportation

simulation was 180 ms.

Table S4: Reactions and rate constants used in 2D Comsol model. M, N and P present neutrals, R represents reactant ion, MR, NR and PR present product ions.

Reaction	Forward rate constant ($cm^{-3} \cdot s^{-1}$)	Reverse rate constant ($cm^{-3} \cdot s^{-1}$)
$M + R \rightarrow MR$	$kr_M = 1.39 \times 10^{-9}$	-
$N + R \rightarrow NR$	$kr_N = kr_M$	-
$P + R \rightarrow PR$	$kr_P = 0.5 \times kr_M$	-
$NR + M \leftrightarrow N + MR$	$kr_{CR} = 0.8 \times kr_M$	$0.1 \times k_{CR}$
$PR + M \leftrightarrow P + MR$	kr_{CR}	$0.1 \times k_{CR}$
$PR + N \leftrightarrow P + NR$	$kr_{CR2} = 0.04 \times kr_M$	$0.1 \times k_{CR2}$

The model and detailed parameters are available on request.

Study of sample injection via GC-capillary split valve into a Stop Flow Confined Volume

Based on the simulation (see Figure S10), it seems that in a certain configuration of electric fields, the reactant ion beam can be focused inside the drift tube, and therefore the sample distribution of the sample injection can affect the response. To understand the magnitude of this effect, the sample introduction was studied with flow modeling. The sample injection was simulated in 2D in the configuration mimicking the GC-capillary spilt valve (Figure S12). A 40 $\mu L \cdot s^{-1}$ sample was injected into the reaction region through 150 μm ID capillary from a needle (shown as a black tube in left of the right pane picture in Figure S12). using a 0.5 s smoothed pulse into the transfer tube, combined with 50 $mL \cdot min^{-1}$ carrier in and 45 $mL \cdot min^{-1}$ carrier out, leaving 5 $\cdot min^{-1}$ as sample carrier into the reaction region. The transfer tube diameter was 4 mm ID and 70 mm long. The sample pulse was formed from a constant sample concentration multiplied by the pulse form function (Figure S12, leftmost pane). The upper and lower sections of the reaction region were defined as 'open' allowing free flux exit from the sample region. The simulation was done in SFCV mode, thus no drift flow was introduced thorough the sample region.

The simulation predicts long-lasting stabilization of the sample in the confined volume in case there is no drift flow. Based on the simulation, the response time may be a function of the stabilization of the distribution of sample concentration in the reaction region. The simulation also predicts that capillary injection may push sample towards the opposite wall, leading to a process of adsorption and desorption. To avoid such risk, the surface quality and materials in the reaction region may be critical and surfaces should be maintained in elevated temperatures.

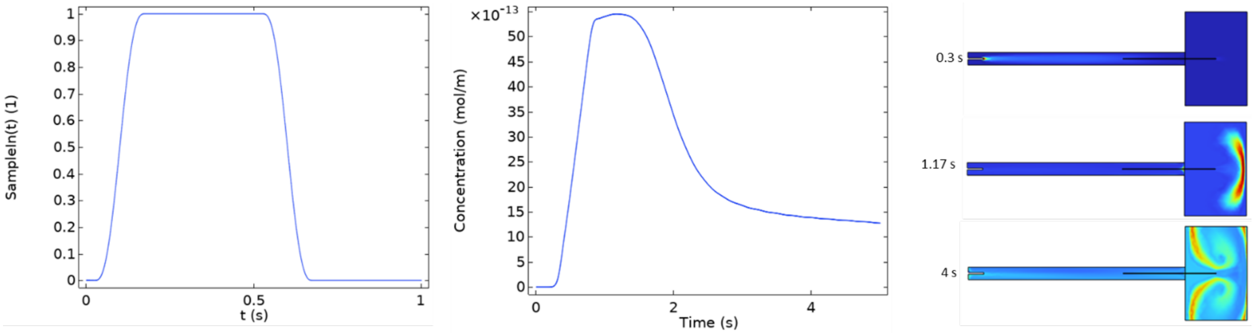

Figure S12: Sample distribution in the reaction region after injection. Left pane: temporal sample injection profile function; Middle pane: average sample concentration in reaction region over time; Right pane: illustration of development of sample concentration distribution in the reaction region at moments 0.3, 1.17 and 4 s. The injection needle is shown left (short, thin black line), and a capillary column connects the transfer line and reaction region (long black line).
